# Dry/Wet Cycling and the Thermodynamics and Kinetics of Prebiotic Polymer Synthesis

**DOI:** 10.3390/life6030028

**Published:** 2016-07-26

**Authors:** David S. Ross, David Deamer

**Affiliations:** 1 Retired, Formerly SRI International, Menlo Park, CA 94025, USA; 2 Department of Biomolecular Engineering, University of California, Santa Cruz, CA 95064, USA; deamer@soe.ucsc.edu

**Keywords:** hydrothermal ponds, RNA, kinetics, thermodynamics, polynucleotides, evaporites, molecular crowding

## Abstract

The endoergic nature of protein and nucleic acid assembly in aqueous media presents two questions that are fundamental to the understanding of life’s origins: (i) how did the polymers arise in an aqueous prebiotic world; and (ii) once formed in some manner, how were they sufficiently persistent to engage in further chemistry. We propose here a quantitative resolution of these issues that evolved from recent accounts in which RNA-like polymers were produced in evaporation/rehydration cycles. The equilibrium N*_m_* + N*_n_* ↔ N*_m+n_* + H_2_O is endoergic by about 3.3 kcal/mol for polynucleotide formation, and the system thus lies far to the left in the starting solutions. Kinetic simulations of the evaporation showed that simple Le Châtelier’s principle shifts were insufficient, but the introduction of oligomer-stabilizing factors of 5–10 kcal/mol both moved the process to the right and respectively boosted and retarded the elongation and hydrolysis rates. Molecular crowding and excluded volume effects in present-day cells yield stabilizing factors of that order, and we argue here that the crowded conditions in the evaporites generate similar effects. Oligomer formation is thus energetically preferred in those settings, but the process is thwarted in each evaporation step as diffusion becomes rate limiting. Rehydration dissipates disordered oligomer clusters in the evaporites, however, and subsequent dry/wet cycling accordingly “ratchets up” the system to an ultimate population of kinetically trappedthermodynamically preferred biopolymers.

## 1. Introduction

A significant conundrum related to the origin of life is the question of how life-relevant polymers overcame the thermodynamic barriers to their formation on an aqueous early Earth in the absence of metabolism and biologically activated monomers [1]. Studies seeking to overcome the barrier with a range of chemical activators have been described [2,3,4,5,6,7,8,9,10], but ultimately they were less than successful in accounting for the prebiotic formation of macromolecules [11]. It is apparent, moreover, that even if a route to biopolymers were uncovered there remains the overriding dilemma of a sustained accumulation of biogenic products and a continuing advance to life in the face of the kinetically preferred hydrolyses back to the monomers. 

The apparent thermodynamic challenge to the production of macromolecules that are both stable and incorporate the complexity required for self-organization emerged from the application of biochemical standard Gibbs energies to the aqueous synthesis of peptide, polynucleotide, and glycosidic links, all of which are endoergic. The values, however, represent the energies of the substances at infinite dilution, and apply only to dilute solutions. Life did not necessarily arise out of dilute bodies of water, and, as will be illustrated here, the thermodynamic challenge to bond formation can be overcome in highly concentrated solutions.

Cycles of hydration and dehydration would have been ubiquitous on the early Earth wherever volcanic land masses emerged from the global ocean. Sea water was presumably salty, perhaps even at higher concentrations than today’s ocean [12], but precipitation would supply the equivalent of distilled water to hydrothermal fields on volcanic islands, producing geological conditions analogous to the geysers and hot springs that are abundant in Iceland. Small pools associated with the hydrothermal fields would undergo continuous cycles of filling and evaporation, and the concentration of the solutes through evaporation was demonstrated in the condensation of glycine [13]. More recently Varfolomeev and colleagues have developed discerning kinetic models of prebiotic molecule synthesis driven by thermal cycling [14].

The focus of the effort here are the findings in studies of nucleotide monomers in simulated hydrothermal pools. They revealed the production of RNA-like oligomers after multiple evaporation/hydration cycles of millimolar solutions of adenosine 5′-monophosphate (AMP) and uridine 5′-monophosphate (UMP) at 85 °C and pH 2.5 over characteristic periods of minutes to hours [15]. The products ranged from 10- to >100-mers, similar to those reported first by Rajamani et al. [16]. Significantly, oligomer synthesis was inefficient if mononucleotides were simply dried, but yields were promoted by the presence of monovalent salts, such as KCl, NaCl and NH_4_Cl [17] or phospholipid [15].

Aligned with those findings are recent accounts suggesting that chemistry in the tightly packed environment in present-day cells can offer insight into life’s origins [18,19,20]. The effects of molecular crowding and excluded volumes in such settings provoke extraordinary thermochemical effects that can enhance association and macromolecular folding rates and equilibria by orders of magnitude [21,22]. The process has been concisely described by Ellis: “*The binding event is favoured because the reduction of excluded volume and the concomitant increase in available volume decrease the total free energy of the solution. Thus, the most favoured state is that which excludes the least volume (with respect) to all the other macromolecules present*”. He continues, noting that for peptides the growing thermodynamic pressure then drives “*the collapse of newly synthesized polypeptide chains into compact functional proteins, … (and) the formation of oligomeric structures such as fibrin, collagen and multienzyme complexes…*” [23].

The exercise described here is based upon our speculation that these large diversions from ideality in cellular cytoplasm are mirrored in, and conceivably derived from, the evaporation and resulting concentration in hydrothermal ponds. We examine that view in a series of kinetic simulations of nucleotide oligomerization in pond environments, taking into account both the simple effects of Le Châtelier’s principle and the developing thermochemical effects of molecular crowding. The exercise bears out the notion that the factors governing the chemistry shift from a setting unfavorable to polymer formation to one that promotes and ultimately sustains it.

## 2. Background and Approach

The prevailing views of the thermochemical and kinetic challenges to the assembly of life-related polymers are largely based on the hydrolytic proclivities of their linking bonds that had been established in dilute aqueous solutions at or near pH 7. A summary of those values is presented in Table 1, as are the Arrhenius parameters for the hydrolysis of the bonds and their half-lives at 25 °C and pH 7.0 and at hydrothermal pond conditions. There are multiple links in any given polymer and their accumulated hydrolysis rates will increasingly limit polymer lifetimes. An RNA-like polynucleotide composed of 100 nucleotides, for example, would display a half-life of no more than about 18 days at 25 °C. The lifetimes at hydrothermal pond conditions would be briefer still, and indeed the sustained existence of nucleotides themselves at those conditions would appear to be problematic because of reactions leading to depurination and deamination.

On the other hand, the three RNA species mRNA, tRNA and rRNA in the crowded cytoplasm of hyperthermophilic microorganisms all survive and their cells thrive in settings approaching 100 °C [29]. The results from both the glycine oligomerization and the hydrothermal pond studies also stand in contrast to the expectation that hydrolysis will overwhelm condensation. The focus of the effort here are the reactions in the latter, the generation of the 2′-5′ and 3′-5′ phosphate-sugar ester bonds linking nucleotide monomers in RNA synthesized in the non-enzymatic condensation reaction in Equation (1).

(1) $$N_{m}+N_{n}⇄N_{m+n}+H_{2}O$$

It would appear initially that evaporative concentration and Le Châtelier’s principle would drive the system to the desired polymer products, but it is easily shown that a simple shift in concentration cannot explain the observations. Employing 3.3 kcal/mol for the formation of phosphate esters at 85 °C, K_1_ ≈ 1.0 × 10^−3^, and leads to required starting monomer concentrations for the dominance of the dimer, N_2_ > N_1_, of an obviously unattainable 300 M. Still greater values would be required for larger oligomers, and it is apparent that additional factors must be in play to bring about the observed formation of oligomers in the pond simulations.

Those factors can be developed with attention to the evident parallel between the highly concentrated evaporation residues and the crowded interiors of present-day cells. Fully 15% of the cell medium is directly associated with a molecular surface [30], and macromolecules can occupy up to 30% of the total cell volume [31]. Further, networks of small channels and cavities within cells induce severe reductions in the activity of water, while the dielectric constant of water declines sharply in the vicinity of the water/organic interface [32]. All of these factors ultimately lead to an energetic condition favoring molecular association, and thereby supporting ester formation in Equation (1).

A measure of that support can be developed from the expression for the free energy of Equation (1) in terms of the activities of the components [33]:

(2) $$\Delta \text{G}\prime =\Delta G^{{}^{\circ}}\prime +\text{RTln}\frac{a_{N_{m+n}}}{a_{N_{m}}a_{N_{n}}}a_{W}$$

At the high dilution the second term is effectively unity, ΔG′ = ΔG^o^’, and thus the equilibrium lies well to the left. The nucleotide activity fraction falls over several decades with crowding, however, and with increasing concentrations of larger product molecules it begins to shape the expression, ultimately reducing ΔG′ by several kcal/mol [23]. Indeed a fundamental feature of the process is the progressive enabling of association reactions with increasing levels of crowding through the fact that the magnitude of the effect grows with the degrees of association [34].

**Figure 1:**
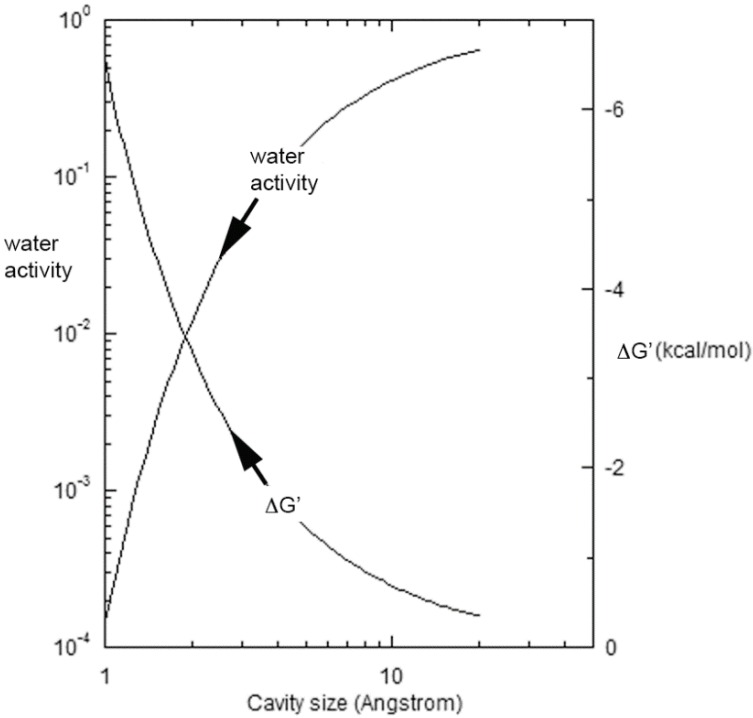
The activity of water and the corresponding Gibbs energy benefit driving the equilibrium in Equation (1) to the right at 85 °C as a function of the characteristic cavity size.

A second source of a reduction in ΔG′ is the decline in the activity of water with the growing population of confining spaces in the residue. The effects can be approximated by a form of the Kelvin equation:

(3) $${\text{ln a}}_{w}=-\frac{2{\text{λV}}_{m}}{\text{dRT}}$$

where λ and V_m_ are respectively the surface tension and molar volume of water at temperature T, d is the characteristic size of the cavity, and R is the universal gas constant [35]. The sharp decline in *a*_w_ with diminishing cavity size is shown on the left for 85 °C in Figure 1, while the right-hand ordinate displays the corresponding Gibbs energy benefit driving the equilibrium in Equation (1) to the right. Toppozini et al. found the thickness of 5′-adenosine monophosphate layers within ordering lipid matrices to be about 2.7 Å [36], and the figure shows that cavities and channels on that order would provide an additional 2–3 kcal/mol of stabilization. Additional stability factors of 4–5 kcal/mol would derive from base pairing and duplex formation [37,38,39], or yet still greater levels as discussed below that can develop with secondary structure. Overall, the effects of evaporation supporting oligomer formation can sum to 10–15 kcal/mol, leading rapidly to a pronounced production of polynucleotides as is described next.

## 3. Results

### 3.1. Evaporation

A selection of values for ΔG′ was employed in a series of numerical simulations of the shifts of the equilibria in Equation (1) at 85 °C. The exercise was carried out for all values of *m* and *n* up to the 50-mer employing the Kintecus simulation software package for complex chemical systems [40], operating on a sequence of 1225 equilibria that was generated with a simple qbasic code. We settled on the 50-mer endpoint for both practical and operational reasons, and as will be seen below the simulations yielded both a crossover point at which oligomer formation became favored and a final equilibrium state. Ideally such systems with no designated upper n-mer will endlessly approach but never attain crossover nor equilibrium (The authors are grateful to Paul Higgs for constructive comments on this topic and for his providing and an advanced copy of a manuscript submitted for publication). Realistically however, limiting factors including declining oligomer solubilities and decreasing rates of diffusion will ultimately restrain growth, and it appears that the 50-mer-topped sequence employed here is sufficiently extended to reveal the underlying dynamics and trends of the process. Key to the simulations were kinetic data developed in studies of the hydrolysis of uridylyl (3′-5′) uridine at 90 °C over the pH range 0–12 [28]. The hydrolysis displayed both acid and base catalysis with a minimum rate at pH 5, and with a value of 1.0 × 10^−6^·s^−1^ at pH = 2.5. This rate constant, k_−1_, served as the foundation of our simulations and as a simplifying parameter applied to all of the hydrolysis steps. When applied to the condensation steps, the rate of the synthesis reaction is then k_−1_ K_1,_ with K_1_ = e^−ΔG′/RT^ (It was crucial to employ a known rate constant in the simulations, and the studies of Oivanen et al. [28] quite suitably provided a value for k_−1_. Thus k_1_ was the adjustable parameter. Accordingly while in the text we refer to the applied ΔG′ values as product-oligomer stabilizing, in truth this treatment equivalently destabilizes the Equation (1) reactants. This operation does not affect the reaction dynamics, however, and the trends displayed in the overall result correctly reflect the sway of product stabilization).

The simulations were conducted in two phases, starting first with the evaporite itself with ΔG′ = ΔG°′, and then examining the effects of the introduction of a series of declining ΔG′ values. In the experimental work the dry/wet cycles were initiated with the evaporation over 30 min of 0.1 mL of a solution 3.3 mM each in AMP (MW 347 Da) and UMP (MW 324 Da). The densities of the solid monomers are ~2.3 g/mL, and thus the starting volume of solid was ~100 nL. Presuming the residues to be about the same volume, we can then estimate that evaporation reduced the volume by a factor of about 1400, which then established the starting point for the simulations of an equivalent monomer concentration of ~9 M.

**Figure 2:**
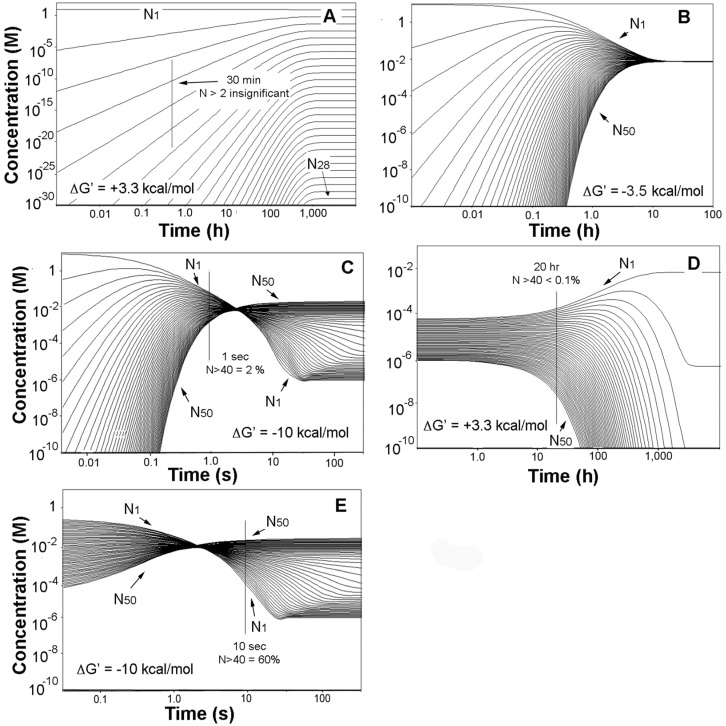
Numerical simulations of the evaporation and rehydration of 6.6 mM solutions of mononucleotide. (**A**) Starting solution concentrated by a factor of 1400 and ΔG′ = ΔG°′; (**B**) products from A at 30 min and ΔG′ = −3.5 kcal/mol; (**C**) products from A at 30 min and ΔG′ = −10 kcal/mol; (**D**) products at 1 s in C diluted by a factor of 1400 and ΔG′ = ΔG°′; (**E**) products at 20 h in (**D)** concentrated by a factor of 1400 and ΔG′ = −10 kcal/mol. The 30-min mark in (**A)** is nominally the point at which the evaporations were complete.

Simulation of the evaporation alone yielded the family of profiles shown in Figure 2A. Consistent with the earlier statement that a simple Le Châtelier’s principle approach to oligomerization was insufficient, the figure shows that the monomer with an overall concentration decrease of no more than 15% remains as the overwhelmingly dominant substance in the mixture, which moreover requires about 1000 h to attain equilibrium.

The thermochemical effects of crowding were then introduced, and we arbitrarily selected the product mixture at the 30-min mark in Figure 2A as the starting point. The behavior of the system shifted markedly in two ways; the product mixtures were largely compressed and ultimately inverted, and the times to equilibrium were radically condensed. The effects are shown in Figure 2B for ΔG′ = −3.5 kcal/mol, for which case the product profiles have all coalesced to about the same concentration while the time to equilibrium fell to about 10 h. The result of a −10 kcal/mol stability factor is displayed in Figure 2C and shows that the system has in this case moved to an oligomer growth region where elongation is energetically favored, and each oligomer in the evaporite is thus more stable than its immediate precursors. Moreover the time to equilibrium has now fallen to a few tens of seconds, reflecting substantial kinetics adjustments to the mounting thermodynamic favoring of oligomer production.

### 3.2. Rehydration, Disaggregation, and Kinetic Traps

Oligomer formation is not boundless, however. Diffusion rates of biomolecules at the conditions within living cells have been shown to fall exponentially with the concentration of crowding agent [22,23,41], and similar effects are expected to develop during evaporation. Insertion of the kinetic effects of diffusion limits into our simulations is beyond the scope of the development here, but polymerization boundaries and the asymptotic approach of polymerization to equilibrium models are well described [42]. They ultimately become rate limiting, and oligomerization ceases at that at point in the present case. The overall description of the reaction course to product thus includes movement along a path first promoted by favoring thermodynamic factors induced by molecular crowding, but then stalled by the congestion that the crowding introduces.

It is of course the case that successive dry/wet cycling leads to improved oligomer yields [17], and thus molecular aggregation and obstruction are not fatal features of the process and appear to be surmounted by cycling. Rehydration will clearly return diffusion rates to their initial high values; however at first glance we would expect that the product oligomers would then revert to the monomer state as rapidly as they had been formed, and there would be no net benefit to cycling. Significantly, however, oligomerization and hydrolysis are respectively second and first order kinetically, and as a result dilution back to the starting condition largely inflates the interval to the monomer-dominated equilibrium. The behavior was seen in the simulations in Figure 2, and can be understood on a quantitative basis by considering any step of Equation (1) in terms of the expression for opposing bimolecular and unimolecular reactions described by Laidler [43].

(4) $$\text{τ}=\;\frac{1}{(a_{m}^{eq}+a_{n}^{eq})k_{1}+k_{-1}\;}$$

Here, τ is the period required for the reaction to navigate the fraction 1/*e* (~37%) of its path to equilibrium, and the activity terms refer to values at equilibrium (This approach is most often employed in kinetic studies of rapid reactions in which τ, referred to as the relaxation time, reflects the time to recovery from abrupt perturbations displacing the system from equilibrium). The form of the expression plainly reveals the large shift Equation (1) will display in its shuttling between dilute and concentrated states. For highly dilute solutions τ is effectively 1/k_−1_, or on the order of hundreds of hours at pond conditions. In contrast with evaporation, sizable solute concentrations will largely increase the value of the k_1_ term which will then dominate the denominator, and τ will fall by similar increments. The gap can span several orders of magnitude as is apparent in Figure 2A–C, and accordingly serves as the foundation of the oligomer-growth effects of dehydration/hydration cycling yields in such systems.

Two factors thus emerge to direct the behavior of the product mixture during cycling. As the sequence starts the oligomeric products rapidly attain some fraction of a thermodynamically favored state during evaporation, but at some point their growth becomes diffusion limit-restrained. Upon dilution the protracted hydrolysis rates trap the products kinetically, but at the same time molecular aggregation is relieved and the disordered oligomer clusters are dissipated and positioned to renewed ester formation in the following dehydration step. Successive rounds of cycling then bring about a step-wise energetic descent to a population of thermodynamically favored oligonucleotides. 

The advance of this progression is illustrated, starting with Figure 2C and assuming that the process becomes diffusion rate-limited at the 1-s mark as shown. The system at that point is then static, with no more than a small fraction of the monomer converted to 40- to 50-mers. The product mixture is then diluted back to its original volume in Figure 2D, initiating hydrolysis. But as is apparent in the figure the process is slow with the time to equilibrium drawn out to thousands of hours. After some period of time—we arbitrarily select a 20-hour interval in the figure—the 40- to 50-mer content has fallen by more than a factor of ten, and a second evaporation is introduced. The result seen in Figure 2E shows that the system quickly moves to a point well beyond its position prior to the dilution, and moreover beyond the energy equivalence point and into the oligomer-growth regime in no more than seconds. A successive series of these events will then yield a stable population of oligonucleotides as was noted above. Natural conditions conforming to such a sequence would include the vigorous splashing of hot springs and geysers onto rock surfaces heated to near boiling temperatures and leading to very rapid rates of evaporation. More generally and depending on their size, typical pools in hydrothermal sites evaporate on time scales of hours to days and are then refilled by precipitation or geyser activity, and still more brief wet-dry cycles occur around the edges of pools due to fluctuating water levels [44]. 

## 4. Discussion

The overall picture that emerges here is one of a series of dry/wet cycles, engaging in alternating rapid oligomer synthesis and slow oligomer hydrolysis while mediated by diffusion limits, in the end yielding a population of thermodynamically favored oligonucleotides. The ponderous nature of the process and a reflection of the effects of diffusion limits early in the progression were exemplified in the experimental work where the first cycle yielded little more than dimers, trimers and tetramers [17]. Longer strands developed over ensuing cycles, and the addition of monovalent salts, such as NaCl, KCl and NH_4_Cl, promoted the process. The extensive polymerization in the end is most reasonably rationalized through the organization of the monomer and smaller oligonucleotides into linear arrays that then undergo ester bond formation in the last stages of drying.

### 4.1. Polymer Synthesis and the RNA World Scenario

The results described here can be applied directly to some of the concerns that at present curb confidence in the RNA World scenario for life’s origin [45]. One such issue is the matter of the hydrolytic viability of RNA and an ongoing search for routes to stabilized intermediates that lead to useful products and avoid fruitless tar-forming reactions [46]. As we have argued here, however, a thermodynamically-directed oligomerization provides the necessary stabilization as a matter of course, with increasing populations of polymers as crowding develops through successive cycles of evaporation. In the end the sole kinetic factor is the rate differential for the respective evaporite and dilute states, which as shown here could amount to orders of magnitude favoring oligomer synthesis. On this basis, the question is not whether intermediates leading to RNA can be stabilized. Rather, the precondition is a setting that provides stability to the intermediates themselves such that their reactions to still more productive products can compete kinetically with a throng of nonproductive and degradative processes. Evaporative venues and the resulting excluded volume conditions appear to deliver that environment.

The observations presented here are also relevant to the problems associated with the view that the sequences provided by the assembly reactions were random; it seems clear, however, that a thermodynamically-driven sequence would be markedly nonrandom and stability-based. Some of the problems related to product randomness were discussed by Robertson and Joyce who described the challenge of sufficient numbers of a self-replicating ribozyme for replication to take place [45]. They referred to the need for 2 copies of a self-replicating RNA in a collection of random sequence 40-mers, and concluded that the magnitude of the required quantity of RNA would be comparable to the mass of the Earth. We can add that the extent of the problem is heightened when the time required for the encounter of the two molecules is considered. For a 1 M solution of 40-mer (presumably in a suitable planet-sized container) and the theoretical maximum encounter constant of 10^9^ M^−1^·s^−1^ [47], the characteristic encounter time is about 10^7^ years. An activation energy of no more than 1–2 kcal/mol would extend the time for reaction by another order of magnitude, while more dilute solutions would require still greater reaction periods. Issues of randomness in reactants vanish, however, with a polymerization with some thermodynamic guidance. Although the extent of thermochemical differentiation within RNA sequences is unknown [48], it is undoubtedly in place to some degree since Gibbs energies can be quite sensitive to structure. A simple example of the sensitivity to structure is reflected in the five hexane isomers, the Gibbs energies for which at 25 °C in the gas phase span more than 2 kcal/mol [49]. If we assume that one of the 40-mer sequences is a ribozyme catalyst with no more than a 1 cal/mol benefit over the other sequences, for example, 1 milligram of a mixture of 40-mers would then contain no less than 10^14^ copies of that molecule. 

An energy gap on that order, however, pales with respect to the potential stability available to sequences that can develop secondary structure. Helix P18 in Escherichia coli RNase P RNA, for example, is a 20-mer, one of 10^12^ possible sequences [50], stabilized through base pairing by about 11 kcal/mol [51]. Such factors fundamentally ensure a significant population of far fewer sequences in accord with the observations of Higgs who has shown that tRNAs are considerably more stable than comparable random sequences [52]. In actual fact, the operation of a fully thermodynamically driven (i.e., wholly reversible) oligomer assembly may not be required for directed product formation. Kinetic control alone can serve to guide product direction based upon linear free energy relationships relating the free energies of the transition states for a series of like reactions to a linear combination of the energies of the respective reactants and products [53]. Thus if some fraction of the stabilizing features in helix P18, 4–5 kcal/mol for example, were reflected in the energy of the transition state leading to that sequence, its rate of formation would then exceed that of most of the other sequences by more than 3 orders of magnitude.

### 4.2. Competition between Synthetic and Degradative Processes

Although synthesis of ester bonds linking mononucleotides into oligomers has been demonstrated experimentally, competing degradative reactions must be taken into account. Mungi and Rajamani, for example, observed significant levels of depurination in hydrothermal simulations at pHs below 3.0 [11]. Carbohydrates are subject to chemical damage through the Maillard reaction and caramelisation, producing the familiar brown polymer recognized in baked and grilled food. In the specific case of nucleotide polymerization, the question turns to whether chemical decomposition of ribose takes place at rates competitive with those of polymer formation at hydrothermal conditions, and on that basis Larralde et al. ruled out sugars as prebiotic reagents [54]. Their study included the decomposition of ribose at elevated temperatures in media ranging from pH 7.5 down to pH 4.5 and revealed that although the rate of the base-catalyzed process declined over that span, the falloff reversed at still lower pHs as the governing process shifted to its acid-catalyzed complement.

That portion of the process was only discussed on a qualitative basis, but their findings are nonetheless useful here in developing some sense of the lifetimes of free ribose in hydrothermal pools at elevated temperature ranges. Their data suggest half-lives of ribose at 80 °C of about 1.3 days at pH 7 increases to about 3.4 years at pH 4.5. If we then presume that the acid catalyzed component becomes controlling at just that point, the lifetime at still lower pHs will decline by factors of ~10^4.5−pH^, so that at pH 2.5 its half-life falls to about 20 h. Notably such periods are significantly greater than the characteristic times for ester formation observed in the laboratory simulations. Moreover the Larralde et al. study examined ribose in free solution, but incorporation of ribose into polymers is likely to markedly reduce acid-catalyzed decomposition. From this, we conclude that ribose-based polynucleotide synthesis in acidic hydrothermal pools is tenable.

Duplex formation can be an additional stabilizing factor countering degradative reactions in single-stranded polymers. Both non-enzymatic phosphoester hydrolysis [55] and cytosine deamination [56] are reduced in double stranded polymers by factors up to 100-fold. Further, it is notable that hyperchromicity of the polymer products was reported by De Guzman et al. [15] and Da Silva et al. [17] suggesting that the single stranded products contain some fraction of duplex strands, probably in the form of hairpin structures.

## 5. Conclusions

The notion of excluded volume-enhanced kinetic and thermodynamic drivers to stable biopolymers in evaporating systems paired with their being kinetically trapped in their subsequent hydrolyses provides a coherent and surprisingly rapid path to the earliest functional biomolecules. The scheme eliminates the inhibiting elements of randomness and chance in their assembly, and since further functional organization and ultimate self-replication require continued molecular growth and folding, which are themselves volume-reducing reactions, the proposition can be applied further along the path leading to higher complex and composite biomolecular systems. Hypercyclic schemes and the broader class of autocatalytic sets, for example, have been described as potential routes to molecular reproduction and ultimately to the emergence and abundance of functional RNA [57,58]. The elements are beyond the scope of the discussion here, but it is noteworthy that the vital components of these networks are molecular templates that must cycle continually within reproduction sequences engaging in second- and third-order reactions. The effectiveness and viability of the template network are challenged by kinetically competitive first-order hydrolyses, and thus an essential feature of a productive prebiotic autocatalytic set would be a dominating augmentation of the association kinetics. As described here, cycling dehydrating/hydrating settings appropriately provide that boost.

## Figures and Tables

**Table 1:** Thermochemical and kinetic parameters governing the formation and hydrolysis of biochemical linking functionalities.

Thermochemical and Kinetic Factors		Protein, Amide	Polysacch., Glycoside	RNA, Phosphate Ester	DNA, Phosphate Ester
ΔG°′ formation/kcal/mol ^a^		2.2	3.8	3.3	
Arrhenius parameters, hydrolysis, pH 7 ^b^	log A/year^−1^	15.5	16.0	10.5	14.1
	E_a_/kcal·mol^−1^	25.2	31.5	15.6	26.8
Hydrolytic half-lives, linking functionality	25 °C, pH 7	385 years	4 M years	5 years	0.2 M years
	85 °C, pH 2.5	7 min ^c^	250 h ^c^	8 days ^d^	876 years ^e^

^a^ Standard biochemical Gibbs energies, taken from Table 13-4 in Nelson and Cox (2005) [24]; ^b^ developed from data for pH 7 in Wolfenden and Snider (2001) [25]; ^c^ the hydrolyses of amides (Mabey and Mill, 1978) [26], and *O*-glycosides (Wolfenden and Yuan, 2008) [27] are acid catalyzed; these values are accordingly boosted by a factor of 10^(7−2.5)^; ^d^ From the data of Oivanen et al. for the hydrolysis of uridylyl (3′-5′) uridine at pH 2.5 and 90 °C (1998) [28]; ^e^ estimated from the fractional change in RNA in the shift from 25 °C/pH 7 to 85 °C/pH 2.5.
